# A Case Series Analysis of Hospital Volume in a New Era of US Bariatric Surgery within the Nationwide Readmissions Database

**DOI:** 10.1007/s11695-025-08300-x

**Published:** 2025-10-04

**Authors:** Zachary Leslie, Sean Nguyen, David Leishman, Sayeed Ikramuddin, Eric Wise

**Affiliations:** 1 Carleton College, Northfield, United States; 2 Department of Surgery, University of Minnesota Medical Center, Minneapolis, United States

**Keywords:** Bariatric surgery, Hospital volume, Readmission, Morbidity

## Abstract

**Background:**

Bariatric surgery is an effective treatment for class III obesity, and higher hospital volume is associated with improved outcomes. We examined readmission, morbidity, and length of stay (LOS) by bariatric center volume in the Nationwide Readmissions Database (NRD).

**Methods:**

The NRD from 2016 to 2022 was used to identify VSG and RYGB procedures with each hospital stratified by very low (1–24), low (25–49), medium (50–124), and high (≥ 125) annual case volume. Univariate tests assessed differences for each volume stratum. 90-day readmission and morbidity were modeled using multivariable logistic regression; LOS was analyzed with a random intercepts model for unique hospitals.

**Results:**

Overall, 4.6%, 6.9%, 31.1%, and 57.6% of patients underwent surgery at very low, low, medium, and high volume centers. Patients at lower volume centers had lower socioeconomic status and higher risk profiles. Readmission and morbidity rates were 15.4%, 6.9%, 4.1%, and 3.2% and 26.0%, 13.2%, 11.9%, and 10.9%, respectively for each volume designation. Very low volume centers showed the highest risk, with low and medium volumes also significantly associated with increased readmission and morbidity. For LOS, low volume had the highest estimated increase of all predictors with medium volume centers also predicting increased LOS. Individual hospitals represented a significant source of variation for total LOS.

**Conclusions:**

Although patients who had surgery at lower volume centers had higher risk profiles, after adjustment there is evidence that lower volume centers are more often associated with increased adverse events. Healthcare teams, accreditation centers, and insurance providers should strive to design and follow patterns of care associated with lower risk.

**Supplementary Information:**

The online version contains supplementary material available at 10.1007/s11695-025-08300-x.

## Introduction

Class II obesity and greater, defined as body mass index (BMI) ≥ 35 kg/m^2^, affects roughly 30% of the US population and is associated with multiple comorbidities that negatively impact both health and quality of life [1, 2]. Bariatric surgery remains the most effective treatment against obesity, and vertical sleeve gastrectomy (VSG) and Roux-en-Y gastric bypass (RYGB) are the most commonly performed procedures. These operations are generally of low overall risk, and result in significant excess weight loss within the first year [3]. Although readmissions and complications are uncommon, they contribute to higher healthcare costs and worse outcomes for affected patients when they do occur [4]. Data from the Metabolic and Bariatric Surgery Accreditation and Quality Improvement Program (MBSAQIP) database, mostly US data, suggests 30-day readmission rates of 5% and major surgical morbidity of 3% [5, 6]. Bariatric surgery is most frequently performed in high volume settings by surgeons with high procedural volumes. Likely, surgeon teams at each hospital improve patient management with the repetitions and familiarization this higher volume affords [7].

The largest accreditation system (MBSAQIP) for bariatric surgery in the USA was started in 2012, and aims to improve quality and safety of bariatric surgical care with a single national standard [8]. As part of this system, the MBSAQIP accredits both low acuity centers (25 or more annual cases) and comprehensive centers (50 or more annual cases) after achievement of benchmark clinical outcomes and other necessary criteria, and these hospital volume ranges suggest that they are essential measures of quality outcomes for bariatric patients. However, the MBSAQIP annual dataset does not provide center designations, and this makes center volume outcomes impossible to decipher [8]. Additionally, it only provides data on 30-day outcomes which is a limitation of the MBSAQIP. Studies analyzing separate datasets have differed on bariatric outcomes stratified by hospital volume. The last nationally representative US study of hospital volume through the Nationwide Inpatient Sample (NIS) in 2005 determined that low volume bariatric surgery could be safe and effective and was not substantially different from higher volume bariatric surgery [9]. This was also corroborated by other regional studies around the same time period [10, 11]. Moreover, there has been no national level re-examination of hospital volume outcomes since VSG became the most prominent bariatric procedure a decade previously [12].

The Nationwide Readmissions Database (NRD) is a nationally representative weighted sample of US hospitalizations that includes Internation Classification of Diseases, 10th revision (ICD-10) codes and standard clinical and demographic variables. Readmissions are tracked within individual calendar years using unique admission-linking identifiers. The relationship between hospital volume and bariatric surgery has not previously been explored in the NRD and is impossible to explore in the MBSAQIP dataset [11]. This study aims to identify the impact of hospital volume in bariatric centers on 90-day readmission and morbidity as well as length of stay (LOS) after the VSG and RYGB bariatric procedures in the NRD from 2016 to 2022.

## Methods

### Data Source

The Healthcare Cost and Utilization Project (HCUP) NRD core and hospital files were combined from 2016 to 2022. The NRD dataset contains over 120 million inpatient visits connected by unique identifiers from 30 state databases which are weighted to be nationally representative. The 10th revision of the international statistical classification of diseases and related health problems (ICD-10-PCS for procedures; ICD-10-CM for diagnoses) was used to identify VSG and RYGB procedures performed in the first nine months of each year to determine true 90-day complication rates; this cohort was stratified using the total number of VSG or RYGB procedures for each unique hospital identifier into very low (1–24 cases/year), low (25–49 cases/year) and medium (50–124 cases/year) and high (125 + cases/year) volume hospitals. Inclusion and exclusion criteria are outlined in a CONSORT diagram (Supplementary Fig. 1). This study has been deemed exempt from IRB review because of the de-identified nature of the dataset used. This analysis adheres to the STROBE guidelines for reporting of large cohort studies.

### Preoperative and Perioperative Factors

Age and biological sex, insurance-payer status, and year of operation were gathered from variable names in the NRD. Residential income and city size were included as the variables for median residential zip code income—coded between 1 and 4 with 1 as the lowest income zip code of residence and 4 as the highest income zip code for patients—and county size of residence for the patient, coded from 1 as the largest city to 6 as the smallest city [13]. Individual hospital bed size and procedural volume characteristics were extracted from the NRD hospital file. Hospital categories were defined as very low volume (1–24), low volume (25–49), medium volume (50–124) and high volume (≥ 125) as defined by prior literature [8]. Currently, the MBSAQIP grants comprehensive (> 49) and low-acuity (> 24) center accreditation. Medical diagnoses with high prevalence or significance for bariatric surgery were identified using appropriate ICD-10-CM codes and tabulated (see all ICD-10-CM and ICD-10-PCS codes in Supplementary Table 1 and Supplementary Table 2). Common baseline comorbidities in patients who undergo bariatric surgery were included as well as healing factors for patient recovery.

### Postoperative Factors

Readmission and morbidity after 90 days were chosen as the outcomes of choice in this study. Readmission rates were calculated as any second admission occurring within 90 days of initial discharge, and only patients with an initial admission in the first nine months of the year had a calculated outcomes rate because the NRD cuts off at the end of each calendar year. Morbidity within 90 days of procedure was identified by designated ICD-10 codes (Supplementary Table 2). A 90-day morbidity event was defined as any (at least one) of these complications occurring in an initial or subsequent admission within 90 days of the initial operation. Length of stay (LOS) was also included from a data field in the NRD.

### Statistical Analysis

Inside the NRD, four population strata based on the hospital categories were compared with weighted chi-squared tests for categorical variables and Kruskal–Wallis tests for continuous variables. Normality was assumed by the central limit theorem and large sample size available in this dataset. Categorical variables in the characteristic table were represented as a percentage of the total while continuous variables were represented as mean ± standard deviation. A propensity matched analysis for higher volume (aggregated as high and medium volume) against lower volume (low and very low volume centers) was performed as well for matching variables of age, sex, and Charlson comorbidity indexes. Multivariable testing was necessary because low, medium, and high volume centers serve patient populations with different risk factors. Multivariable binomial regression models were created adjusting for baseline demographic, preoperative, hospital, and comorbidities for each patient to determine the risk of low and medium volume bariatric centers for the binary dependent variables 90-day readmission and morbidity. A Bonferroni-adjusted *p* < 0.001 was deemed statistically significant when comparing the three cohorts, and a *p* < 0.001 was used to determine significance for each individual factor in the multivariable binomial logistic regression model. Additionally, a linear mixed effect model was created for the continuous dependent variable LOS to determine the effect each unique hospital had as a random effect which was modeled with random intercepts for each unique hospital. For all tables with multivariable analysis, cells were represented as odds ratio (OR) [95% confidence interval] (CI) or coefficients (Coeff) [95% CI] for the categorical and continuous variable, respectively. The random effect in the mixed linear effects model is represented as a variance [95% confidence interval]. Python (v. 3.12) and the pandas, polars, statsmodels, and scikit-learn libraries were used for all analyses.

## Results

### Demographics

A weighted total of 732,405 patients were identified from the first 9 months of 2016–2022, with 4.6%, 6.9%, and 31.1%, and 57.6% coming from very low, low, medium, and high volume hospitals, respectively (Table 1). More frequently (*p* < 0.001) in lower volume centers patients were residents of the state where the operation was performed, Medicare payers, older, from smaller cities/towns, and of lower income status. Patients at higher volume centers were significantly more female, private insurance payers, from larger cities, and of higher income status. There is no association with Medicaid status and hospital volume.

**Table 1:** Baseline characteristics, stratified by very low (1–24 cases/year), low (25–49 cases/year), medium (50–124 cases/year), and high volume bariatric centers (125 + cases/year) using the Nationwide Readmissions Database from 2016 to 2022. Kruskal–Wallis and weighted chi-squared tests for three populations used to assess significance across the cohort

Variable	Very low volume (4090 hospitals, 33,639 cases)	Low volume (744 hospitals, 50,497 cases)	Medium volume (1481 hospitals, 227,432 cases)	High volume (1069 hospitals, 420,836 cases)	*p*-value
Demographics					
Resident	96.42%	96.05%	94.61%	95.25%	< 0.001
Female sex	79.87%	82.32%	81.68%	81.15%	< 0.001
Medicare	23.17%	17.14%	14.74%	11.01%	< 0.001
Medicaid	22.71%	22.89%	22.04%	22.85%	< 0.001
Private insurance	46.69%	51.94%	56.5%	59.89%	< 0.001
Age	45.46 (± 12.76)	44.83 (± 12.09)	44.28 (± 12.06)	43.16 (± 11.91)	< 0.001
Large city/metropolitan area	45.78%	43.72%	49.72%	63.27%	< 0.001
Small city/micropolitan area	36.94%	41.53%	36.54%	28.15%	< 0.001
Small town, not micropolitan or metropolitan	17.24%	14.74%	13.73%	8.57%	< 0.001
Low income	28.59%	25.3%	26.29%	24.7%	< 0.001
Medium income	58.39%	55.64%	54.39%	50.45%	< 0.001
High income	16.83%	17.3%	19.32%	22.26%	< 0.001
Hospital/operative factors					
Small bed size	26.84%	21.65%	16.54%	11.78%	< 0.001
Medium bed size	24.8%	30.00%	28.94%	22.37%	< 0.001
Large bed size	34.93%	35.17%	41.42%	51.75%	< 0.001
2016	13.79%	13.74%	12.84%	13.02%	< 0.001
2017	14.0%	13.35%	14.3%	16.02%	< 0.001
2018	11.97%	12.25%	11.63%	13.97%	< 0.001
2019	15.89%	13.54%	15.53%	17.22%	< 0.001
2020	16.25%	18.11%	16.56%	9.49%	< 0.001
2021	14.66%	15.84%	16.05%	16.19%	< 0.001
2022	13.42%	13.17%	13.09%	14.1%	< 0.001
2022	13.42%	13.17%	13.09%	14.1%	< 0.001
2022	13.42%	13.17%	13.09%	14.1%	< 0.001
2022	13.42%	13.17%	13.09%	14.1%	< 0.001
2022	13.42%	13.17%	13.09%	14.1%	< 0.001
Roux-en-Y gastric bypass	14.79%	26.6%	29.24%	29.42%	< 0.001
Government, non federal hospital	8.61%	10.69%	6.77%	8.52%	< 0.001
Private, not-profit hospital	57.14%	60.93%	67.7%	68.57%	< 0.001
Private, invest-own hospital	20.82%	15.21%	12.44%	8.81%	< 0.001
Metropolitan non-teaching hospital	32.33%	26.5%	18.64%	14.42%	< 0.001
Metropolitan teaching hospital	44.81%	54.35%	64.34%	70.2%	< 0.001
Non-metropolitan hospital	9.44%	5.98%	3.93%	1.28%	< 0.001
Comorbidities					
Charlson comorbidity index	1.03 (± 1.37)	0.79 (± 1.09)	0.82 (± 1.11)	0.79 (± 1.06)	< 0.001
Type 2 diabetes mellitus	32.33%	27.51%	27.24%	25.53%	< 0.001
Hypertension	46.94%	48.47%	48.38%	45.18%	< 0.001
Hyperlipidemia	32.35%	32.5%	33.54%	32.29%	< 0.001
Gastroesophageal reflux disease	35.84%	44.17%	43.22%	43.77%	< 0.001
Obstructive sleep apnea	39.05%	45.08%	44.68%	43.21%	< 0.001
Chronic obstructive pulmonary disease	4.24%	2.91%	2.82%	2.1%	< 0.001
Chronic kidney disease	5.84%	3.04%	3.08%	2.54%	< 0.001
Coronary heart disease	5.56%	3.69%	3.36%	3.00%	< 0.001
Chronic heart failure	5.89%	2.58%	2.5%	2.02%	< 0.001
Peripheral arterial disease	0.57%	0.26%	0.27%	0.26%	< 0.001
Healing factors					
Long-term NSAID	7.58%	5.54%	5.34%	4.52%	< 0.001
Alcohol abuse	0.38%	0.13%	0.14%	0.17%	< 0.001
Liver disease	0.18%	0.18%	0.19%	0.18%	0.40
Long term use steroids	0.78%	0.35%	0.36%	0.30%	< 0.001

*Long-term NSAID*, Long-term Non-steroidal anti-inflammatory drugs

### Hospital/Operative Factors

There were 4090, 744, and 1481, and 1069 very low, low, medium, and high volume hospitals, respectively. Patients from lower volume centers were significantly (*p* < 0.001) more often at small bed size hospitals, private investment owned hospitals, and metropolitan non-teaching hospitals as well as non-metropolitan hospitals. Patients which had an operation performed at higher volume centers significantly more frequently were at larger bed size centers, private non-profit hospitals, and metropolitan teaching hospitals.

### Comorbidities and Healing Factors

Very low volume center patients had higher average Charlson Comorbidity Index values relative to patients with operations performed at higher volume counterparts (very low: 1.0 ± 1.4 vs. low: 0.8 ± 1.1 vs. medium: 0.8 ± 1.1vs. high: 0.8 ± 1.1). Overall, lower volume hospital patients had higher rates of more severe comorbidities which include chronic kidney disease (CKD), coronary heart disease, chronic heart failure (CHF), and peripheral arterial disease. They also had higher rates of type 2 diabetes, obstructive sleep apnea, long-term non-steroidal anti-inflammatory drugs (long-term NSAID), alcohol abuse, and long-term usage of steroids.

### Univariate Outcome Analysis

**Fig. 1:**
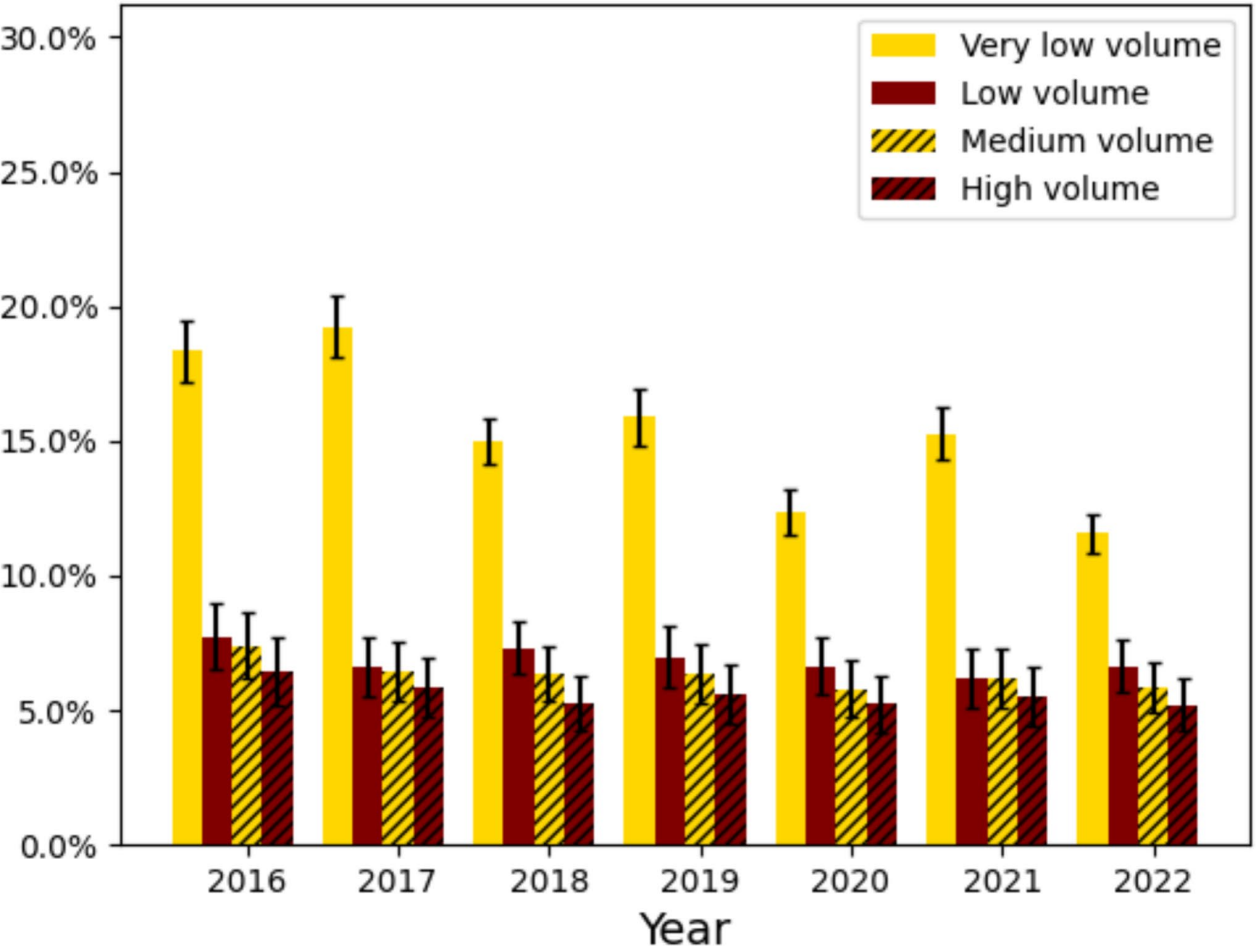
Rates of 90-day readmission stratified by hospital volume in the first 9 months of the Nationwide Readmissions Database from 2016 to 2022 for patients who had a vertical sleeve gastrectomy or Roux-en-Y gastric bypass performed

**Fig. 2:**
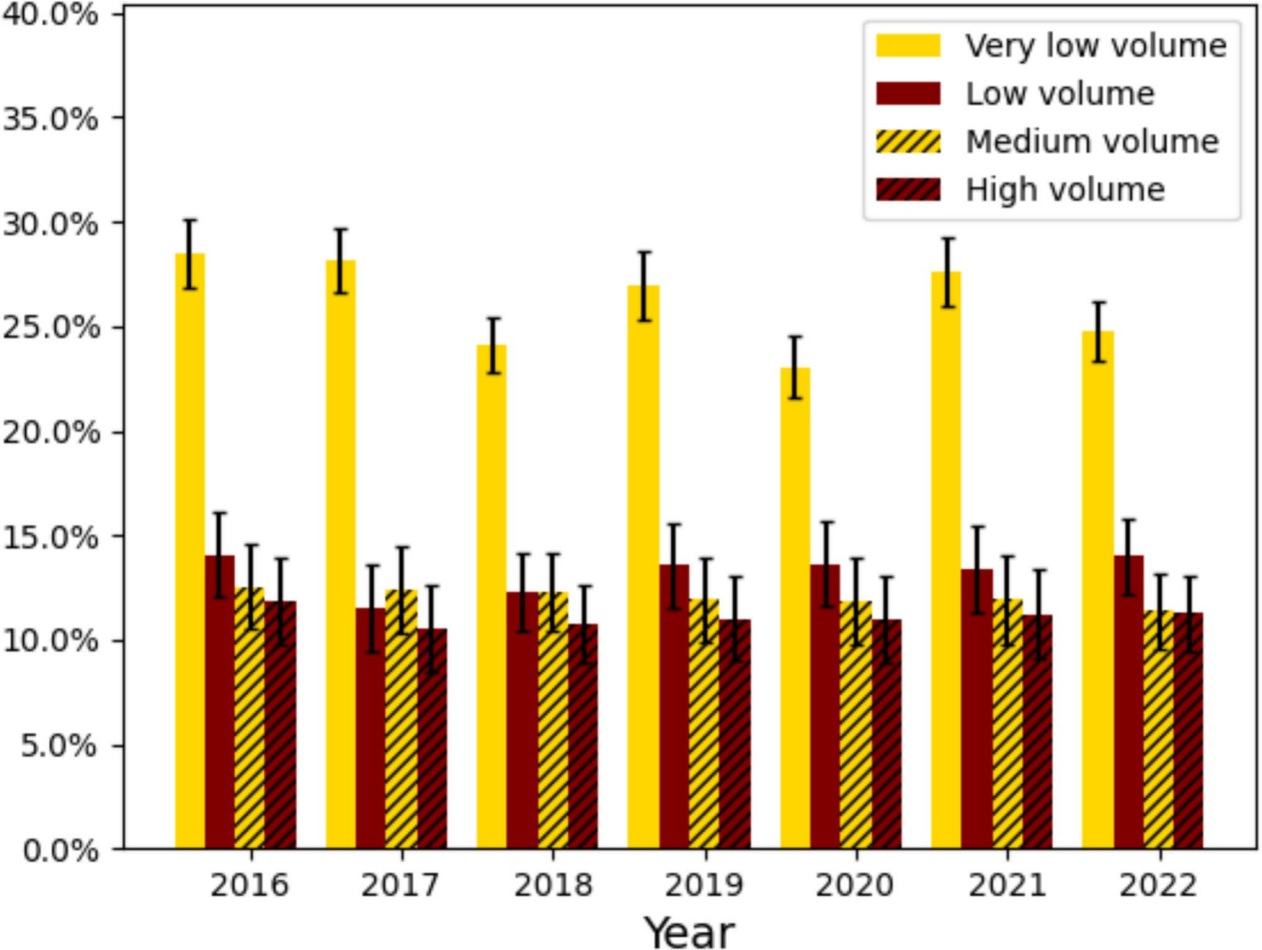
Rates of 90-day morbidity stratified by hospital volume in the first 9 months of the Nationwide Readmissions Database from 2016 to 2022 for patients who had a vertical sleeve gastrectomy or Roux-en-Y gastric bypass performed

There were significant differences in rates of 90-day readmission and morbidity across patients at low, medium, and high volume centers. 90-day readmission rates were 15.4%, 6.9%, 4.1%, and 3.2% across very low, low, medium, and high volume bariatric centers. 90-day morbidity rates were 26.0%, 13.2%, 11.9%, and 10.9%, and across very low, low, medium, and high volume centers (Table 2, Figs. 1 and 2). Overall, the most common complications were electrolyte derangements (very low: 13.1% vs. low: 6.0% vs. medium: 5.2% vs. high: 5.0%), cardiac arrhythmias (very low: 6.0% vs. low: 3.4% vs. medium: 3.1% vs. high: 2.6%), and infections (very low: 5.1% vs. low: 2.1% vs. medium: 1.7% vs. high: 1.3%). Additionally, the total charges were on average lower for higher volume hospitals. Propensity matching analysis on age, sex, and Charlson comorbidity index consistently shows higher rates of adverse events in lower volume groups (Supplementary Table 3).

**Table 2:** Outcomes stratified by very low (1–24 cases/year), low (25–49 cases/year), medium (50–124 cases/year), and high volume bariatric centers (125 + cases/year) using the Nationwide Readmissions Database from 2016 to 2022. Kruskal–Wallis and weighted chi-squared tests for three populations used to assess significance across the cohort

Variable	Very low volume (4090 hospitals, 33,639 cases)	Low volume (744 hospitals, 50,497 cases)	Medium volume (1481 hospitals, 227,432 cases)	High volume (1069 hospitals, 420,836 cases)	*p*-value
Total charges	55,516.03 (± 49,910.23)	57,547.44 (± 40,659.34)	56,289.74 (± 41,825.81)	53,640.35 (± 37,526.48)	< 0.001
Fragmentation of care within 90 days	34.14%	5.15%	4.13%	3.23%	< 0.001
Mortality	0.01%	0.04%	0.02%	0.02%	0.10
90-day readmission	15.36%	6.85%	6.3%	5.56%	< 0.001
90-day morbidity	25.94%	13.21%	11.87%	10.88%	< 0.001
LOS	2.29 (± 2.62)	1.76 (± 1.61)	1.69 (± 1.71)	1.71 (± 1.95)	< 0.001
90-day delirium	0.4%	0.18%	0.16%	0.11%	< 0.001
90-day cerebral infarction	0.35%	0.1%	0.09%	0.07%	< 0.001
90-day cardiac arrhythmia	6.03%	3.42%	3.08%	2.64%	< 0.001
90-day cardiac arrest	0.13%	0.09%	0.08%	0.06%	< 0.001
90-day myocardial infarction	0.57%	0.19%	0.19%	0.14%	< 0.001
90-day heart failure	0.0%	0.0%	0.0%	0.0%	0.72
90-day other cardiac	0.33%	0.38%	0.3%	0.28%	0.00
90-day ventilator dependency	0.89%	0.39%	0.39%	0.31%	< 0.001
90-day infection	5.1%	2.07%	1.71%	1.27%	< 0.001
90-day pneumonia	4.98%	1.2%	0.88%	0.61%	< 0.001
90-day wound issues/SSI	0.69%	0.45%	0.41%	0.3%	< 0.001
90-day shock	0.82%	0.49%	0.49%	0.36%	< 0.001
90-day bleeding	0.59%	0.5%	0.54%	0.46%	< 0.001
90-day blood transfusion	2.0%	1.06%	0.91%	0.91%	< 0.001
90-day PE	0.99%	0.4%	0.38%	0.29%	< 0.001
90-day DVT	0.87%	0.38%	0.38%	0.32%	< 0.001
90-day acute renal failure	0.05%	0.04%	0.02%	0.02%	< 0.001
90-day complications	0.2%	0.1%	0.1%	0.08%	< 0.001
90-day derangements	13.14%	5.98%	5.22%	4.98%	< 0.001

*LOS* length of stay, *SSI* surgical site infection, *PE* pulmonary embolism, *DVT* deep vein thrombosis

### Multivariable Outcome Analysis

Demographic factors which confer increased risk of 90-day readmission and morbidity were residence status of the state where the operation was performed, low/middle income status, and increased age. Medicaid status, small city/micropolitan area patient residence, small town patient residence, and private insurance each were associated with decreased risk (Tables 3 and 4). Very low volume bariatric centers (90-day readmission: 2.94 [2.83, 3.06], 90-day morbidity: 2.62 [2.53, 2.70]) was the health factor associated with the largest increase in risk of both 90-day readmission and morbidity. Low and medium volume centers also conferred additional risk (low, 90-day readmission: 1.26 [1.21, 1.32], 90-day morbidity: 1.20 [1.16, 1.24], medium, 90-day readmission: 1.13 [1.10, 1.16], 90-day morbidity: 1.07 [1.05, 1.09]). Small bed size was associated with decreased risk of 90-day readmission and morbidity. Of the 7 fixed effects and 1 random effect analyzed, each was significant (Table 5). Very low volume caseload had the highest estimated increase in LOS (1.03 [0.94, 1.11]) and low and medium volume centers having a not statistically significant estimated LOS increase, (low: 0.07 [− 0.05, 0.2], medium: − 0.007 [− 0.009, 0.010). Charlson comorbidity index (0.134 [0.132,0.136]) also increased the estimated LOS. The random effect, unique hospitals, represented a significant source of variation (1.31 [1.27, 1.36]).

**Table 3:** Logistic regression on dependent variable 90-day readmission NRD 2016–2022 after bariatric surgery

Independent variable	Unadjusted Coeff [95% CI]	Adjusted Coeff [95% CI]	*p*-value
Demographics			
Resident	1.42 [1.35, 1.48]	1.32 [1.25, 1.39]	< 0.001
Female sex	0.91 [0.89, 0.93]	1.01 [0.98, 1.04]	0.38
Medicaid	1.18 [1.16, 1.21]	0.94 [0.91, 0.97]	< 0.001
Private insurance	0.62 [0.61, 0.63]	0.69 [0.68, 0.71]	< 0.001
Age	1.18 [1.17, 1.19]	1.02 [1.01, 1.03]	0
Small city/micropolitan area	0.97 [0.95, 0.99]	0.87 [0.84, 0.89]	< 0.001
Small town, not micropolitan or metropolitan	0.92 [0.89, 0.94]	0.77 [0.74, 0.8]	< 0.001
Low income	1.18 [1.15, 1.2]	1.09 [1.06, 1.12]	< 0.001
Medium income	1.14 [1.12, 1.16]	1.03 [1.01, 1.06]	0.01
Hospital/operative factors			
Small bed size	0.91 [0.89, 0.94]	0.81 [0.79, 0.84]	< 0.001
Medium bed size	1.04 [1.02, 1.06]	0.96 [0.93, 0.98]	0
Year	0.93 [0.92, 0.94]	0.92 [0.91, 0.94]	< 0.001
Very low volume	2.87 [2.79, 2.97]	2.94 [2.83, 3.06]	< 0.001
Low volume	1.09 [1.05, 1.13]	1.26 [1.21, 1.32]	< 0.001
Medium volume	0.99 [0.97, 1.01]	1.13 [1.1, 1.16]	< 0.001
Roux-en-Y gastric bypass	1.29 [1.27, 1.32]	1.37 [1.34, 1.4]	< 0.001
Private, not-profit hospital	0.99 [0.97, 1.01]	1.02 [0.99, 1.06]	0.12
Private, invest-own hospital	1.16 [1.12, 1.19]	1.1 [1.05, 1.15]	< 0.001
Metropolitan non-teaching hospital	1.05 [1.02, 1.08]	0.94 [0.91, 0.96]	< 0.001
Non-metropolitan hospital	1.02 [0.96, 1.07]	0.93 [0.87, 1.0]	0.04
Comorbidities			
Type 2 diabetes	1.48 [1.45, 1.51]	1.14 [1.12, 1.17]	< 0.001
Hypertension	1.07 [1.05, 1.09]	1.14 [1.11, 1.16]	< 0.001
Hyperlipidemia	1.18 [1.16, 1.2]	0.94 [0.91, 0.96]	< 0.001
Gastroesophageal reflux disease	1.11 [1.09, 1.13]	1.07 [1.05, 1.1]	< 0.001
Obstructive sleep apnea	1.05 [1.03, 1.07]	0.9 [0.88, 0.92]	< 0.001
Chronic obstructive pulmonary disease	1.99 [1.9, 2.08]	1.25 [1.18, 1.32]	< 0.001
Chronic kidney disease	3.05 [2.94, 3.16]	2.09 [1.99, 2.19]	< 0.001
Coronary heart disease	2.27 [2.19, 2.36]	1.33 [1.26, 1.39]	< 0.001
Chronic heart failure	3.42 [3.29, 3.56]	2.22 [2.11, 2.33]	< 0.001
Peripheral arterial disease	2.9 [2.58, 3.26]	1.3 [1.13, 1.49]	< 0.001
Healing factors			
Long-term NSAID	1.6 [1.54, 1.65]	1.16 [1.11, 1.21]	< 0.001
Alcohol abuse	1.65 [1.37, 2.0]	1.25 [1.0, 1.56]	0.05
Liver disease	1.2 [0.98, 1.47]	1.13 [0.9, 1.42]	0.29
Long-term use steroids	2.32 [2.07, 2.6]	1.79 [1.57, 2.04]	< 0.001

*Long-term NSAID*, Long-term Non-steroidal anti-inflammatory drugs

**Table 4:** Logistic regression on dependent variable 90-day morbidity NRD 2016–2022 after bariatric surgery

Independent variable	Unadjusted Coeff [95% CI]	Adjusted Coeff [95% CI]	*p*-value
Demographics			
Resident	1.12 [1.08, 1.15]	1.07 [1.03, 1.11]	< 0.001
Female sex	0.74 [0.73, 0.75]	0.94 [0.92, 0.96]	< 0.001
Medicaid	0.95 [0.93, 0.96]	0.92 [0.9, 0.94]	< 0.001
Private insurance	0.63 [0.62, 0.64]	0.76 [0.74, 0.77]	< 0.001
Age	1.49 [1.48, 1.5]	1.23 [1.22, 1.24]	< 0.001
Small city/micropolitan area	0.95 [0.94, 0.97]	0.85 [0.84, 0.87]	< 0.001
Small town, not micropolitan or metropolitan	1.02 [1.0, 1.04]	0.82 [0.79, 0.84]	< 0.001
Low income	1.12 [1.1, 1.14]	1.07 [1.04, 1.09]	< 0.001
Medium income	1.10 [1.09, 1.12]	1.03 [1.01, 1.05]	0.01
Hospital/operative factors			
Small bed size	0.97 [0.95, 0.99]	0.92 [0.9, 0.95]	< 0.001
Medium bed size	0.97 [0.96, 0.99]	0.96 [0.94, 0.98]	< 0.001
Year	0.99 [0.98, 1.00]	0.99 [0.98, 1.00]	0.01
Very low volume	2.71 [2.65, 2.78]	2.62 [2.53, 2.70]	< 0.001
Low volume	1.10 [1.07, 1.13]	1.20 [1.16, 1.24]	< 0.001
Medium volume	0.98 [0.96, 0.99]	1.07 [1.05, 1.09]	< 0.001
Roux-en-Y gastric bypass	1.17 [1.15, 1.19]	1.21 [1.19, 1.24]	< 0.001
Private, not-profit hospital	0.99 [0.97, 1.00]	0.99 [0.96, 1.01]	0.23
Private, invest-own hospital	0.99 [0.97, 1.01]	0.91 [0.88, 0.94]	< 0.001
Metropolitan non-teaching hospital	1.08 [1.06, 1.10]	1.03 [1.0, 1.05]	0.03
Non-metropolitan hospital	1.04 [1.0, 1.08]	0.96 [0.91, 1.01]	0.09
Comorbidities			
Type 2 diabetes	1.71 [1.69, 1.74]	1.11 [1.09, 1.13]	< 0.001
Hypertension	1.28 [1.27, 1.3]	1.32 [1.3, 1.35]	< 0.001
Hyperlipidemia	1.48 [1.46, 1.5]	0.96 [0.94, 0.98]	< 0.001
Gastroesophageal reflux disease	1.13 [1.11, 1.14]	1.04 [1.02, 1.06]	< 0.001
Obstructive sleep apnea	1.33 [1.32, 1.35]	1.01 [1.00, 1.03]	0.12
Chronic obstructive pulmonary disease	2.49 [2.41, 2.57]	1.21 [1.16, 1.26]	< 0.001
Chronic kidney disease	4.63 [4.5, 4.76]	2.82 [2.72, 2.93]	< 0.001
Coronary heart disease	3.48 [3.38, 3.57]	1.57 [1.52, 1.63]	< 0.001
Chronic heart failure	6.11 [5.93, 6.29]	3.5 [3.37, 3.64]	< 0.001
Peripheral arterial disease	3.57 [3.25, 3.91]	1.13 [1.01, 1.27]	0.03
Healing factors			
Long-term NSAID	2.08 [2.02, 2.13]	1.17 [1.14, 1.21]	< 0.001
Alcohol abuse	1.44 [1.24, 1.68]	1.07 [0.89, 1.29]	0.47
Liver disease	0.91 [0.77, 1.08]	0.80 [0.66, 0.98]	0.03
Long term use steroids	2.26 [2.06, 2.48]	1.78 [1.6, 1.98]	< 0.001

*Long-term NSAID*, Long-term Non-steroidal anti-inflammatory drugs

**Table 5:** Linear mixed effects model on dependent variable length of stay NRD 2016–2022 for major bariatric surgery

Variable	Coeff [95% CI]	*p*-value
Female sex	0.054 [0.42,0.66]	< 0.05
Age (years)	0.0030 [0.0031,0.0032]	< 0.05
Year	− 0.049 [− 0.065, − 0.033]	< 0.05
Charlson comorbidity index	0.134 [0.132,0.136]	< 0.05
Roux-en-Y gastric bypass	0.178 [0.172,0.184]	< 0.05
Very low volume	1.026 [0.940, 1.112]	< 0.05
Low volume	0.066 [− 0.05, − 0.190]	< 0.05
Medium volume	− 0.007 [− 0.085,0.099]	< 0.05
Unique hospital ID	1.313 [1.265,1.361]	< 0.05

## Discussion

In this nationally representative analysis using the NRD, we evaluated the impact of hospital volume on outcomes following major bariatric surgery, specifically VSG and RYGB. Our findings highlight a clear and consistent relationship between hospital procedural volume and patient outcomes—patients treated at very low and low volume centers experienced significantly higher rates of 90-day readmission and postoperative morbidity, even after adjusting for socioeconomic, demographic, and clinical risk factors with multivariable logistic regression models, mixed effects models, and propensity matching.

This study is particularly timely and important as it updates the current understanding of hospital volume and bariatric outcomes in the context of modern practice and the MBSAQIP accreditation system. It is also the first national-level study in the USA to assess this relationship since VSG became the predominant bariatric procedure. Our analysis challenges the conclusions of earlier work, including the 2005 NIS study that suggested low-volume bariatric surgery could be equally safe and effective [9]. While our results do not rule out the possibility that certain low-volume centers may provide excellent care, the overall trend is clear: lower hospital volume is strongly associated with worse outcomes, which is mostly driven by very low volume centers. Our results also confirm findings from large meta-analyses, which have shown that high-volume centers generally yield better outcomes in bariatric surgery [11, 12]. Specifically, higher volume centers were associated with decreased risk of morbidity in each study. Notably, we found that unique hospital identity contributed the largest source of variability in postoperative length of stay (LOS), supporting the idea that outcomes may be institution-specific rather than uniformly tied to volume alone. Still, on average, patients at very low-volume hospitals had nearly threefold higher readmission rates and double the morbidity rates compared to those at high-volume centers, suggesting that volume is a reliable and actionable surrogate for quality.

Our study adds nuance to the ongoing discussion around access to bariatric surgery for socioeconomically disadvantaged populations. Patients at very low-volume centers were more likely to be older, publicly insured, from lower-income areas, and living in smaller towns or rural regions. While it is commendable that these centers provide access to vulnerable populations, the disproportionately higher complication rates among these patients raise concerns about equity in surgical outcomes. Improving care at low-volume centers—or directing patients to higher-volume institutions—may be necessary to reduce disparities in postoperative risk.

Interestingly, our study identified higher overall morbidity rates than typically reported in bariatric literature [14, 15]. This is likely due to our broader definition of morbidity, which included electrolyte imbalances, cardiac arrhythmias, and infections—conditions that may not always be captured or emphasized in prior studies [16]. These complications, while potentially less severe than anastomotic leaks, hemorrhage, or venous thromboembolism as examples, are clinically meaningful and may reflect differences in access to postoperative support such as nutritional counseling or outpatient monitoring [13, 17, 18]. Importantly, the most severe complications also remained significantly more common in lower-volume centers. From a policy and accreditation standpoint, these findings are significant. Organizations such as MBSAQIP, insurance providers, and health systems may benefit from using volume thresholds and hospital-specific data as part of quality assurance and referral decision-making. Our results offer strong evidence against policies that encourage bariatric surgery at very low-volume hospitals solely to increase access [11, 12]. Any access-related benefits must be balanced against the demonstrably higher risk of adverse outcomes.

### Limitations

This study has several limitations. As a retrospective, observational analysis, causal inference is limited despite our use of multivariable modelings. The use of ICD-10 codes, while standard, may be subject to misclassification or variability in coding practices, particularly where billing incentives influence documentation. Additionally, the NRD does not provide information on key variables such as years of surgical experience, operative complexity, or preoperative lab values—each of which may further explain outcome variability. The MBSAQIP measures important lab values, operation complexity through minutes the operation takes to perform, and other variables unmeasured by the NRD. Which is why it is preferred by a majority of bariatric surgeons. Lastly, the NRD captures only data from individual calendar years and cannot account for mortality or complications occurring outside of readmissions within the same calendar year. Despite these limitations, this is the largest and most current national study evaluating hospital volume and bariatric surgery outcomes using a modern, real-world US dataset. It provides robust evidence on a topic of ongoing clinical and policy importance.

## Conclusions

This study demonstrates that bariatric surgery performed at very low- and low-volume centers is, on average, associated with significantly worse postoperative outcomes, including higher 90-day readmission and morbidity rates. While some low-volume centers may provide high-quality care, volume remains a reliable and meaningful predictor of outcomes at the population level. Given the high variability in outcomes among hospitals and the increased risk faced by underserved populations often treated at lower-volume centers, additional oversight and support may be warranted. Accreditation bodies, insurers, and policymakers should consider these findings when shaping referral patterns and surgical access frameworks. Future research should include more granular data on surgeon experience, intraoperative variables, and preoperative health metrics to further refine risk stratification and quality improvement efforts.

## Supplementary Information

Below is the link to the electronic supplementary material.

Supplementary file1 (DOCX 77 KB)

## Data Availability

No datasets were generated or analysed during the current study.
