# Supplementary material for: A Case Series Analysis of Hospital Volume in a New Era of US Bariatric Surgery within the Nationwide Readmissions Database
Source: Obes Surg. 2025 Oct 4;35(11):4711–21. doi: 10.1007/s11695-025-08300-x (PMC12594670; doi:10.1007/s11695-025-08300-x)
Supplement: Supplementary file 1 — Supplementary file1 (DOCX 77 KB) [file 11695_2025_8300_MOESM1_ESM.docx]

**Supplementary Information**

Supplementary Table 1: 10th revision of the international classification of diseases – clinical modification (ICD-10 CM) codes and variables from Nationwide Readmissions Database for analysis.

| Variable | ICD-10 Code |
| --- | --- |
| Fibromyalgia | M797 |
| Rheumatoid arthritis | M069 |
| Chronic heart failure | I50, I130, I131, I132, I110 |
| Coronary heart disease | I251 |
| Long term NSAID | Z791, Z7982 |
| Anticoagulant therapy | Z7901, Z902 |
| COPD | J449 |
| Presence coronary graft | Z951, Z955 |
| CKD | N18, I29 |
| Hypertension | I10 |
| Hyperlipidemia | E78 |
| Morbid obesity | E6601 |
| Smoker | F1721 |
| GERD | K21 |
| Type 2 diabetes | E10, E11 |
| B12 anemia | D50, D51, D52, D649 |
| Tobacco nicotine dependence | Z720, F172 |
| Opioid use disorder | F119, F112, F1121, F1120 |
| Cannabis use | F1120, F12988 |
| Anxiety disorder | F410, F411, F418, F419 |
| Dialysis | Z992 |

Supplementary Table 2: 10th revision of the international classification of diseases – procedure coding system (ICD-10 PCS) codes and variables from Nationwide Readmissions Database for analysis.

| Variable | ICD-10 PCS Code |
| --- | --- |
| Roux-en-Y Gastric Bypass | 0D164Z |
| Vertical sleeve gastrectomy | 0DB64Z3 |
| 90-day acute renal failure | N990 |
| 90-day sepsis | R65, A41, B377, P36 |
| 90-day blood transfusion | 302 |
| 90-day UTI | N390 |
| 90-day PE | I82 |
| 90-day DVT | I26 |
| 90-day cardiac arrest | I46 |
| 90-day MI | I21 |
| 90-day infection | A419, T8613, N390, T814XX,  T868, T80211 |
| 90-day respiratory infection | A084, T857, B349, J069,  T827, T835, T8743, |
|  | T80212, T8469, T835, N995,  B348, T8579, T802 |
| 90-day ventilator dependency | 5A1935Z, 5A1945Z, 5A1955Z |
| 90-day pneumonia | J15, J18 |
| 90-day delirium | F05, R400, R401, R4020,  R403, R404, R4182 |
| 90-day cerebral infarction | I63, I97811, I97821 |
| 90-day cardiac arrhythmia | I44, I480, I489, I49 |
| 90-day cardiac Arrest | I46, I97121 |
| 90-day myocardial infarction | I21 |
| 90-day heart failure | I97111, I97131 |
| 90-day other cardiac event | I97191, I973, I9789 |
| 90-day arterial insufficiency | T81718A, T8172XA, T81710A,  T81711A |
| Ventilator dependency | 5A1935Z, 5A1945Z, 5A1955Z |
| 90-day pneumothorax | J95811 J95812 |
| 90-day post operative complications (general) | K913, K9130, K9131, K9132, K917, K9171,  K9172, K918, K9181, K9182, K9183, K91872,  K91873, K9189, K940, K941, K942,  K943,  K950, K958 |
| 90-day infection | K6811, N300, N309, N390, R6520, R6521,  R7881, T8112XA, T8140XA, T8141XA,  T8142XA, T8143XA, T8144XA, T8149XA |
| 90-day pneumonia | A0103, A0222, A202, A212, A221, A310,  A3701, A3711, A3781, A3791, A420, A430,  A481, A5484, B012, B052, B0681, B250,  B371, B380, B390, B400, B583, B59, B7781,  J09X1, J1000, J1001, J1008, J1100, J1108,  J12, J13, J14, J15, J16, J17, J18, J95851 |
| 90-day wound issues/SSI | T8130XA, T8131XA, T8132XA, T8183XA,  T8140XA, T8141XA, T8142XA, T8143XA,  T8149XA, L7634 |
| 90-day shock | T8110XA, T8111XA, T8112XA, T8119XA,  R6521, R570, R571, R578, R579 |
| 90-day bleeding | D780, D782, E360, E8981, G973, G975,  I974, I9761, I9762, J9501, J956, J9583, K916, K9184, K9401, K9411, K9421,  K9431, L760, L762, M9681, M9683, N99510, N99520, N99530, N996, N9982 |
| 90-day blood transfusion | 302 |
| 90-day pulmonary embolism | I26 |
| 90-day deep vein thrombosis | I82 |
| 90-day acute renal failure | N990 |
| 90-day electrolyte derangements | E870, E871, E872, E873, E874, E875, E876 |

Supplementary Table 3: Propensity matched analysis of lower volume bariatric surgery relative to higher volume bariatric surgery with a caliper band of 0.1 and 4:1 match higher to lower volume bariatrics in the Nationwide Readmissions Database from 2016 to 2022.

| **Variable** | **Higher volume (n=322205)** | **Lower volume (n=84135)** | **p-value** |
| --- | --- | --- | --- |
| **Variables cohort matched on** | | | |
| Female sex | 262210 (81.38%) | 68445 (81.35%) | 0.16 |
| Age (Years) | 45.18 (+-12.37) | 45.08 (+-12.37) | 0.13 |
| Charlson comorbidity index | 0.88 (+-1.2) | 0.89 (+-1.21) | 0.70 |
| **Outcomes** | | | |
| Mortality | 68 (0.02%) | 24 (0.03%) | 0.50 |
| 90-day readmission | 19468 (6.04%) | 8591 (10.21%) | <0.001 |
| 90-day morbidity | 38700 (12.01%) | 15348 (18.24%) | <0.001 |
| Length of stay | 1.73 (+-1.92) | 1.97 (+-2.09) | <0.001 |
| 90-day delirium | 427 (0.13%) | 228 (0.27%) | <0.001 |
| 90-day cerebral infarction | 290 (0.09%) | 164 (0.19%) | <0.001 |
| 90-day cardiac arrhythmia | 10358 (3.21%) | 3746 (4.45%) | <0.001 |
| 90-day cardiac arrest | 235 (0.07%) | 91 (0.11%) | <0.001 |
| 90-day myocardial infarction | 608 (0.19%) | 290 (0.34%) | <0.001 |
| 90-day other cardiac | 940 (0.29%) | 301 (0.36%) | <0.001 |
| 90-day ventilator dependency | 1231 (0.38%) | 491 (0.58%) | <0.001 |
| 90-day infection | 4974 (1.54%) | 2744 (3.26%) | <0.001 |
| 90-day pneumonia | 2511 (0.78%) | 2263 (2.69%) | <0.001 |
| 90-day wound issues/SSI | 1136 (0.35%) | 459 (0.55%) | <0.001 |
| 90-day shock | 1484 (0.46%) | 524 (0.62%) | <0.001 |
| 90-day bleeding | 1601 (0.5%) | 453 (0.54%) | 0.07 |
| 90-day blood transfusion | 3225 (1.0%) | 1201 (1.43%) | <0.001 |
| 90-day pulmonary embolism | 1073 (0.33%) | 532 (0.63%) | <0.001 |
| 90-day deep vein thrombosis | 1174 (0.36%) | 483 (0.57%) | <0.001 |
| 90-day acute renal failure | 70 (0.02%) | 41 (0.05%) | <0.001 |
| 90-day complications | 281 (0.09%) | 118 (0.14%) | <0.001 |
| 90-day derangements | 17488 (5.43%) | 7411 (8.81%) | <0.001 |

Supplemental figure 1: CONSORT diagram for inclusion and exclusion criteria for cohort of patients in this study as unweighted values from the Nationwide Readmissions Database.


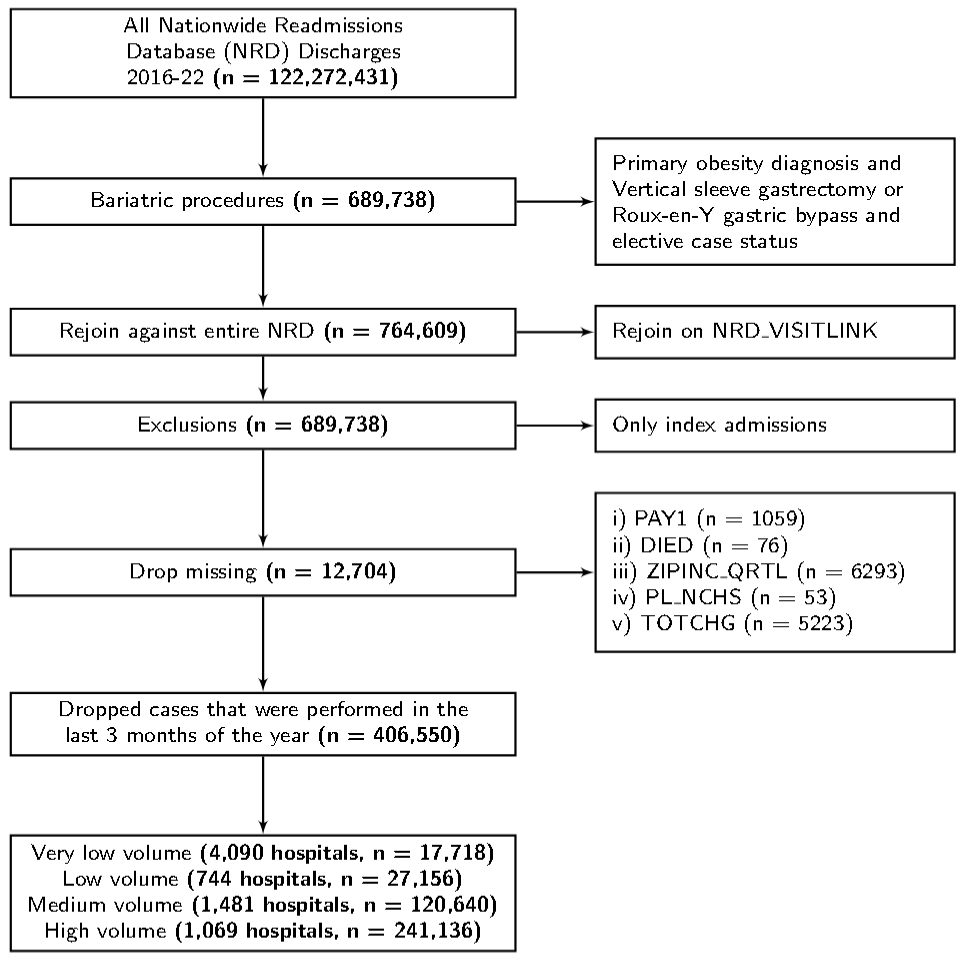


Abbreviations: CONSORT = Consolidated standards of reporting trials, NRD_VISITLINK = unique patient identifier combination, PAY1 = insurance provider information, ZIPINC_QRTL = surrogate for patient income level coded 1-4, PL_NCHS = surrogate for patient city size of residence coded 1-7, TOTCHG = total cost of charges for patient visit.
